# Neuromechanical Adaptation to Walking With Electromechanical Ankle Exoskeletons Under Proportional Myoelectric Control

**DOI:** 10.1109/OJEMB.2023.3288469

**Published:** 2023-06-26

**Authors:** Rachel L. Hybart, Daniel P. Ferris

**Affiliations:** J. Crayton Pruitt Department of Biomedical EngineeringUniversity of Florida 3463 Gainesville FL 32611 USA

**Keywords:** Adaptation, exoskeleton, lower-limb, myoelectric

## Abstract

Objective: To determine if robotic ankle exoskeleton users decrease triceps surae muscle activity when using proportional myoelectric control, we studied healthy young participants walking with commercially available electromechanical ankle exoskeletons (Dephy Exoboot) with a novel controller. The vast majority of robotic lower limb exoskeletons do not have direct neural input from the user which makes adaptation of exoskeleton dynamics based on user intent difficult. Proportional myoelectric control has proven to allow considerable adaptation in muscle activation and gait kinematics in pneumatic, tethered ankle exoskeletons. In this study we quantified the changes in muscle activity and joint biomechanics of twelve participants walking for 30 minutes on a treadmill. Results: The exoskeletons provided 29% of the peak total ankle power and 18% of the peak total ankle moment by the end of the practice session. There was a decrease of 12% in soleus, 17% in lateral gastrocnemius and 5% in medial gastrocnemius electromyography (EMG) root mean square (root mean squared) after walking with the exoskeleton for 30 minutes compared to not wearing the exoskeleton, but this difference was not statistically significant. There were no differences in joint biomechanics of the ankle, hip, or knee between the end of training compared to walking without the exoskeletons. Conclusions: Contrary to expectations, triceps surae muscle activity showed only small non-significant decreases in 30 minutes of walking with portable, electromechanical ankle exoskeletons under proportional myoelectric control. The commercially available ankle exoskeletons were likely too weak to produce a statistically meaningful decline in triceps surae recruitment. Future research should include a wider variety of tasks, including measurements of metabolic energy expenditure, and provide a longer period of adaptation to evaluate the ankle exoskeletons.

## I. Introduction

For robotic exoskeletons to gain widespread consumer acceptance, they need to be more user friendly, mobile and cost-effective [1], [2], [3]. Most robotic exoskeletons that are currently on the market have limitations in their high cost, large weight, and lack of demonstrated efficacy [4]. To be effective, robotic exoskeletons need to provide a metabolic, muscular, and/or biomechanical benefit without increasing the cognitive load on the user [3]. The controller can have a large effect on the efficacy of a robotic exoskeleton independent of the hardware design of the exoskeleton [5], [6], [7], [8]. Most lower limb robotic exoskeleton controllers are based on finite state machines, relying on kinematic and/or kinetic sensors to detect motion patterns and activate the exoskeleton actuators to assist the human's intended movement [2], [9], [10], [11], [12]. These types of controllers can work quite well for cyclic, stereotypical movement patterns like walking at a constant speed.

Proportional myoelectric control provides users with feedforward control of robotic exoskeleton assistance and enables them to improve exoskeleton efficacy with practice [13], [14], [15]. Surface electrodes on the skin can pick up myoelectric signals and convert them to electrical input to the exoskeleton actuators with as few as one EMG sensor per leg. These types of controllers allow for user intention to be considered in the control scheme. This means that the types of movements the device can assist in are not constrained by the ability of the controller to determine the user's state or gait phase. The user can transition from one type of movement to another without the controller changing, and they can adapt their control signal to compensate for fatigue or motor learning.

The goal of this study was to test proportional myoelectric control on an untethered, electromechanical robotic ankle exoskeleton that is commercially available. We implemented soleus proportional myoelectric control of plantar flexion assistance from ExoBoots (Dephy, Inc.) on healthy young participants. We examined muscle activity, gait biomechanics, and exoskeleton performance during a training period of 30 minutes of walking on a treadmill. We hypothesized that triceps surae muscle activation would have lower amplitudes with practice walking compared to walking while not wearing the device. This study follows previous research designs studying healthy, young participants walking with a tethered, pneumatic ankle exoskeleton using a similar controller [5], [16], [17], [18]. In addition to hypothesizing that triceps surae muscle activity would be lower with practice using the exoskeleton, we also hypothesized that the biomechanical values of angle, torque, power, and work at the lower limb joints would not be different after training compared to walking without the exoskeleton [19].

## II. Results

**FIGURE 1.**
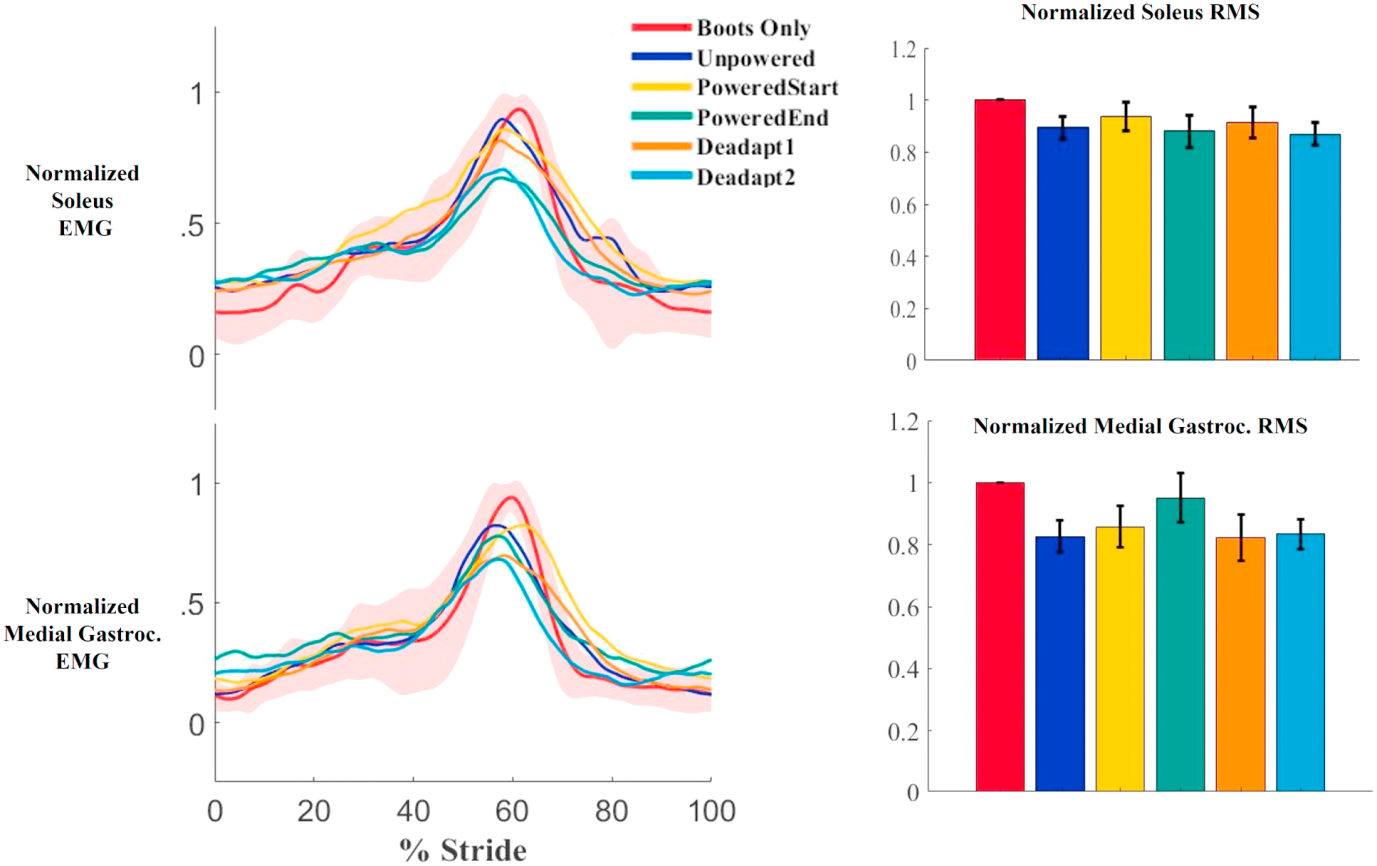
The average EMG activity over the gait cycle (left) and root mean squared (right) of the Soleus and Medial gastrocnemius muscles. Shaded red areas represent ±1 standard deviation. Root mean squared values normalized to the boots only condition for each muscle. Error bars are ±1 s.e.m. In the root mean squared graph, from left to right the conditions are boots only, unpowered, PoweredStart, PoweredEnd, Deadaptation1, and Deadaptation2. Heel contact was at time 0% on the x-axis.

**FIGURE 2.**
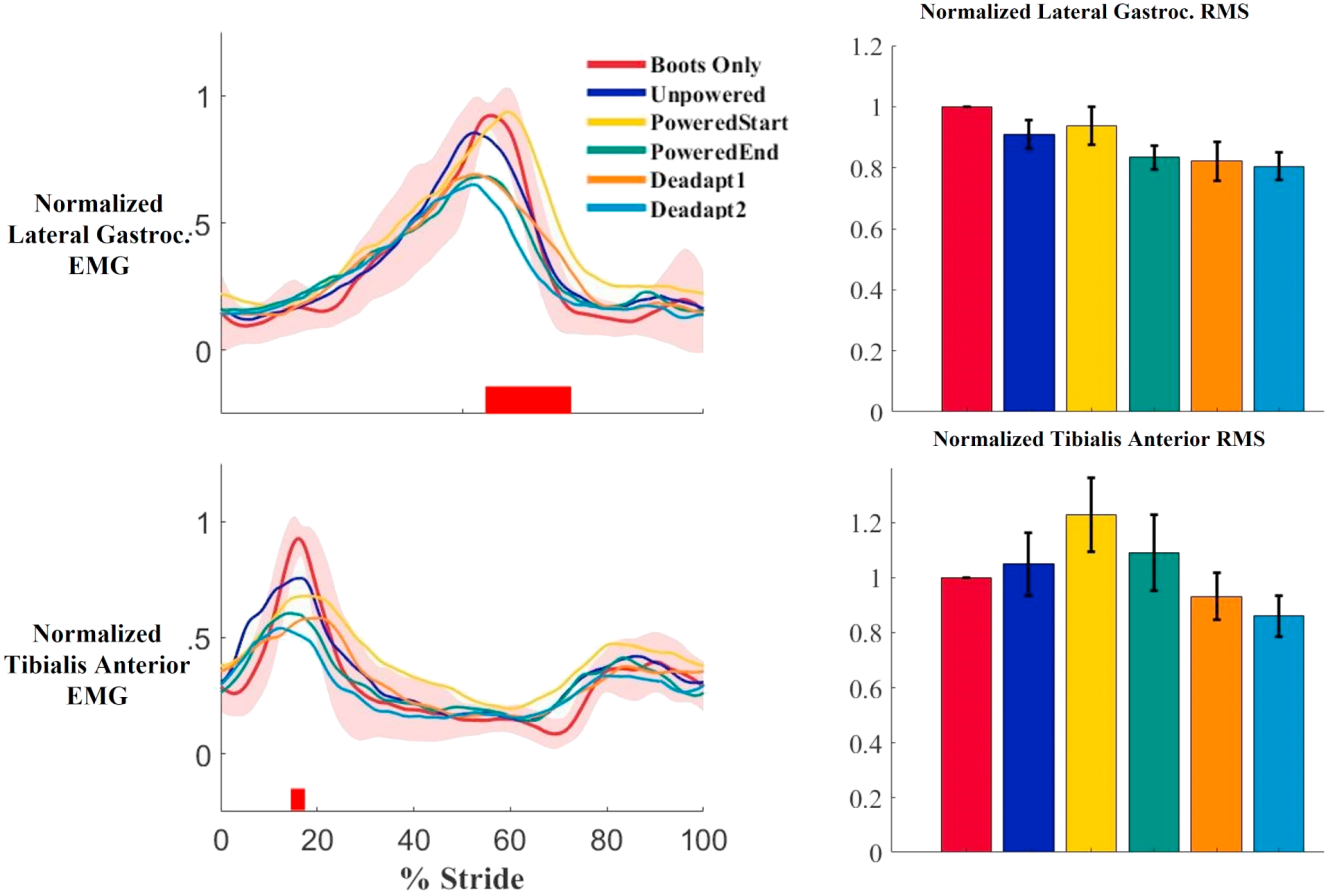
The average EMG activity over the gait cycle (left) and root mean squared (RMS) (right) of the lateral gastrocnemius and tibialis anterior muscles. Shaded red areas represent ±1 standard deviation. Root mean squared values normalized to the boots only condition for each muscle. Error bars are ±1 s.e.m. In the root mean squared graph from left to right the conditions are boots only, unpowered, PoweredStart, PoweredEnd, Deadaptation1, and Deadaptation2. Heel contact was at time 0% on the x-axis. Horizontal red bars indicate portions of the gait cycle where the boots only and PoweredEnd conditions are significantly different (p < 0.05).

Contrary to our hypothesis, there were no significant differences in any of the triceps surae muscles in root mean squared EMG values across the conditions. There were small decreases in muscle activity at the end of the training session compared to the Boots Only condition, but the differences were not statistically significant. Compared to the Boots Only condition, the soleus root mean squared value during stance was 12% less (p = 0.476), the medial gastrocnemius root mean squared value was 5% less (p = 0.168), and the lateral gastrocnemius root mean squared value was 17% less (p = 0.059) (Figs. 1 and 2). Calculation of Standardized Mean Difference values for the three muscles indicates the magnitude of decreases were relatively small (0.12, 0.05, and 0.17, respectively). The tibialis anterior root mean squared during swing was 9% higher at the end of training compared to the Boots Only condition, but the difference was not significant (p = 0.185) (Fig. 2). The Standardized Mean Difference for the tibialis anterior was 0.09.

**FIGURE 3.**
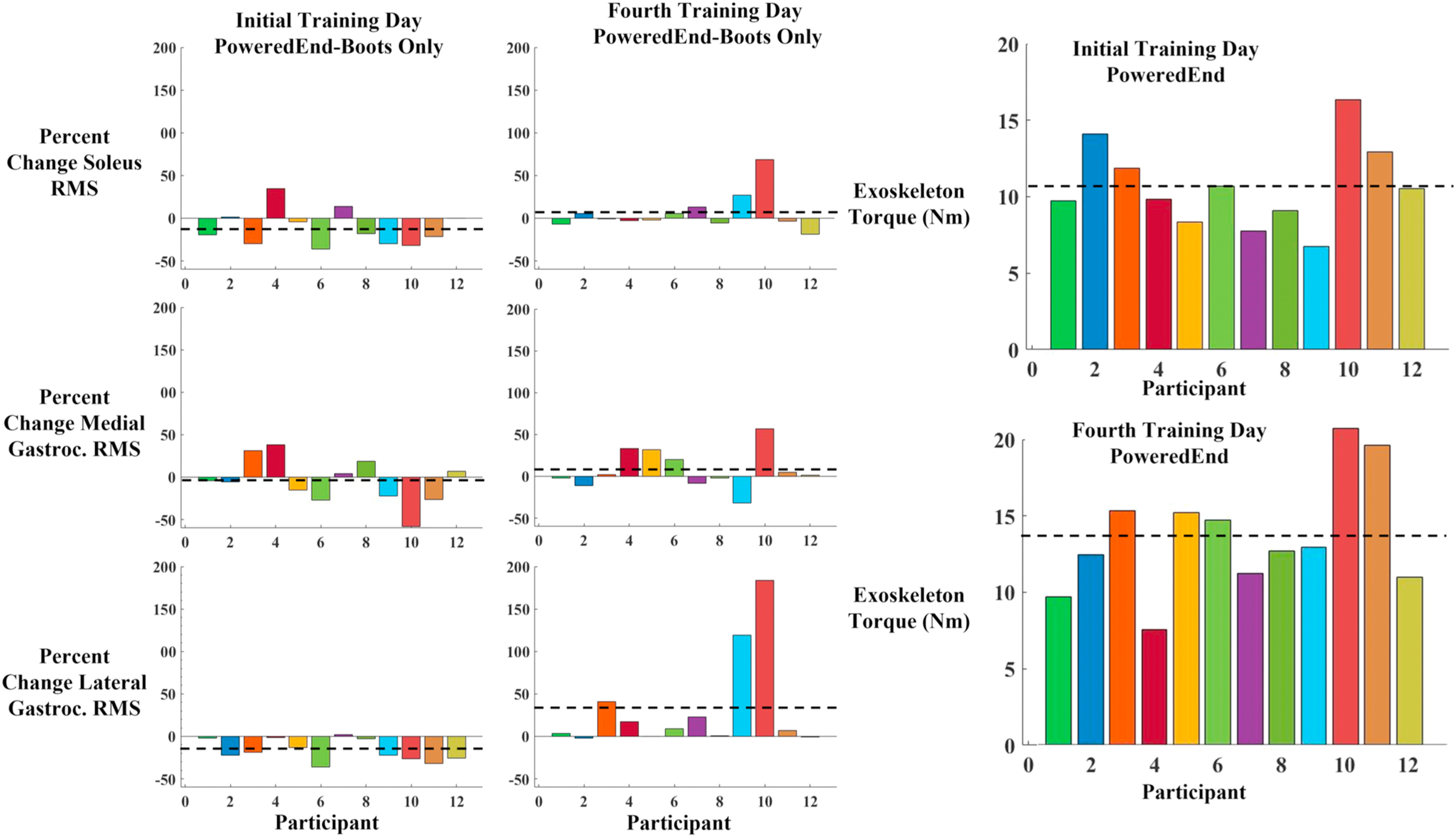
Percent change in EMG RMS between the PoweredEnd and boots only conditions during initial adaptation (left) and after 4 days of training (middle) for the triceps surae muscles for each of the twelve participants. Positive values indicate smaller EMG RMS in the boots only condition and negative values indicate smaller EMG RMS values in the PoweredEnd condition. The exoskeleton torque (Nm) (right) for the powered conditions during initial training and after four days of training for each of the twelve participants. Black dashed lines indicate mean values. The majority of participants reduced their EMG by the PoweredEnd condition during initial training, but not to the same extent after several days of training, but the measures were highly variable. Exoskeleton torque values remained within the same range from initial training to the final day of training, but varied from participant to participant.

There was a large amount of variability in how participant EMG RMS changed due to the exoskeleton. The percent change in soleus RMS from the Boots Only condition to the PoweredEnd condition ranged from −37% to +35%. Negative percent change indicates the RMS was lower in the PoweredEnd condition and positive percent changes indicate that participants had an increase in RMS activation. 67% of participants showed a decrease in soleus RMS in the PoweredEnd condition. The medial gastrocnemius RMS ranged from −55% to +35% across participants. This included 58% of participants having lower medial gastrocnemius RMS in the PoweredEnd condition. The percent change in lateral gastrocnemius RMS ranged from −37% to +2%. The lateral gastrocnemius had the most consistently lower RMS across participants with 92% of participants reducing their lateral gastrocnemius RMS (Fig. 3 left).

On the final day of testing participants showed different EMG RMS values compared to the first day. The percent change in soleus RMS in the PoweredEnd condition compared to the Boots Only condition ranged from −19% to +67%. The medial gastrocnemius RMS percent change ranged from −32% to +55%. The lateral gastrocnemius RMS percent change ranged from 0 to 170% (Fig. 3 middle).

The amount of torque supplied by the exoskeleton across participants was also varied. On the first day of training there was a range of torque values from 7–17 Nm. On the fourth day of training there were torque values from 7–19 Nm (Fig. 3 right).

**FIGURE 4.**
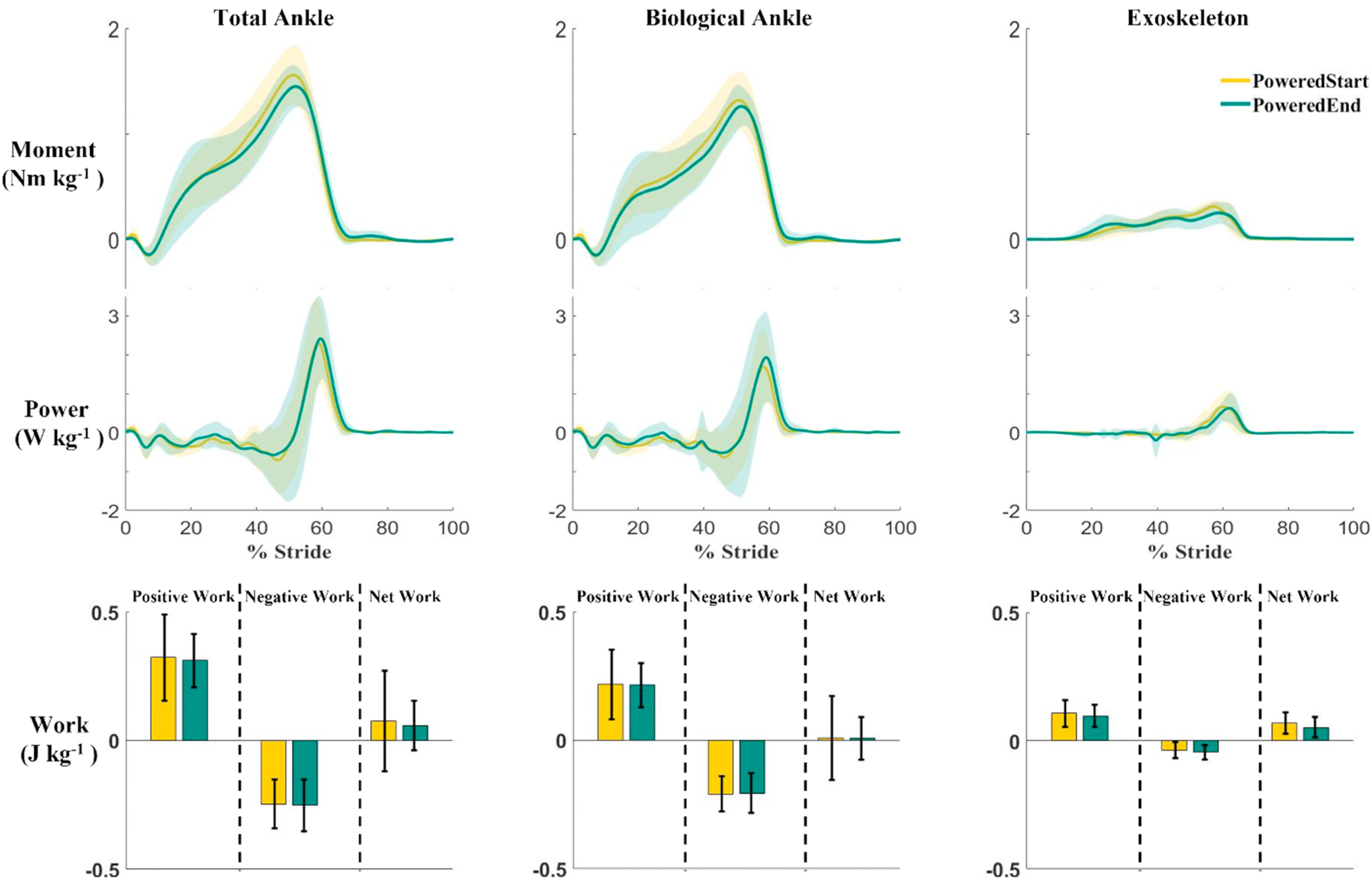
Exoskeleton, biological ankle, and combined average moment, power, and work during the PoweredStart (yellow) and PoweredEnd (green) conditions. Shaded areas at the moment and power plots represent ±1 standard deviation. Black bars in the work plots indicate ±1 standard deviation. Heel contact was at time 0% on the x-axis.

**FIGURE 5.**
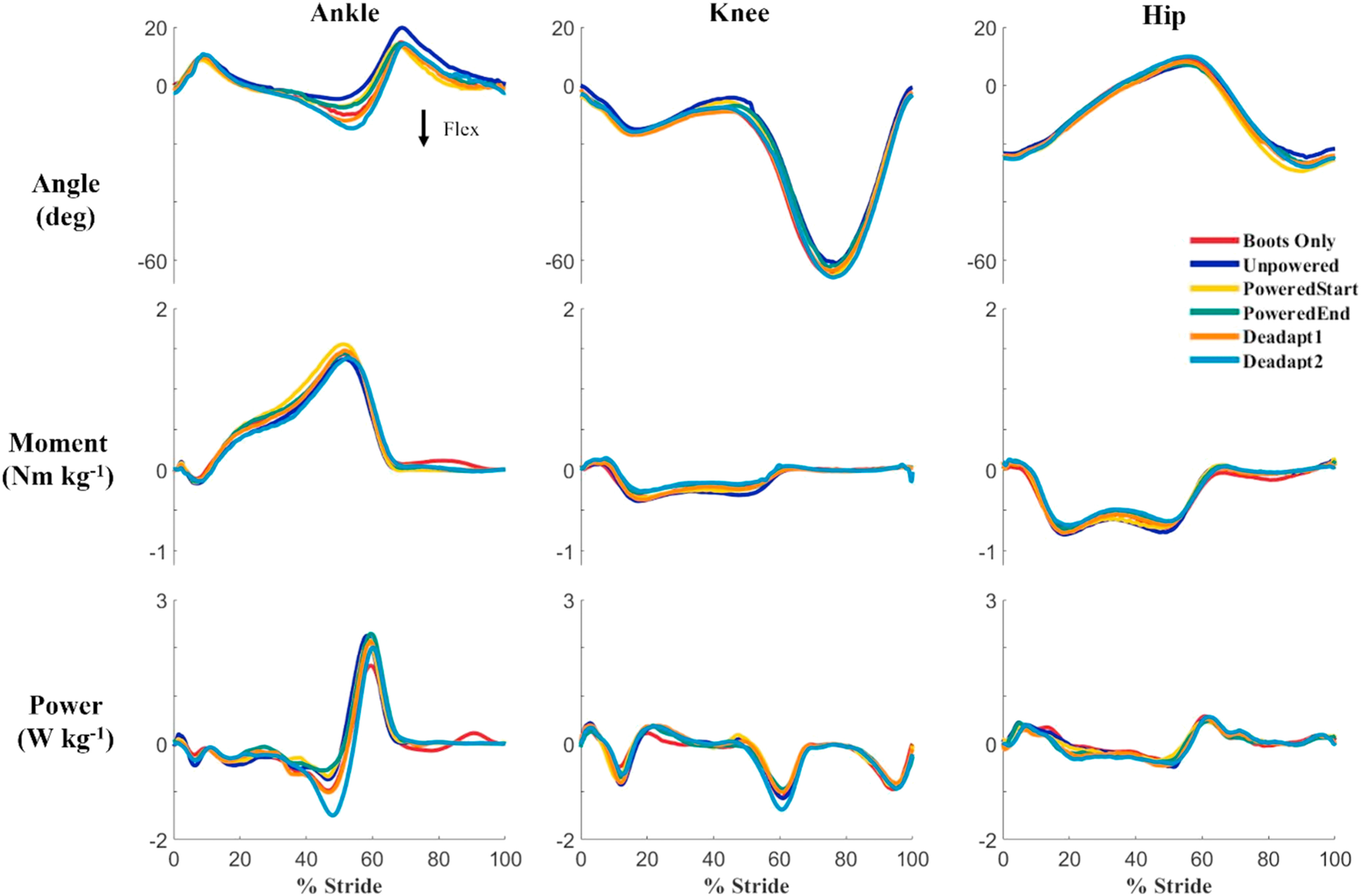
Average joint angle, moment and powers at the ankle, knee, and hip for each condition. There was an average decrease in dorsiflexion of 4 degrees between the powered exoskeleton conditions and the boots only and deadaptation conditions. Peak negative power was highest during the boots only and deadaptation conditions. Knee and Hip joint angles, moments and powers were relatively unchanged between conditions. Heel contact was at time 0% on the x-axis.

The ankle exoskeletons provided considerable mechanical assistance to the users after practice walking. The exoskeletons provided 29% of the peak total ankle power provided by the combined exoskeleton and biological ankle during the PoweredEnd condition. The peak total ankle power during the PoweredEnd condition was 28% higher than during the Boots Only condition. The exoskeletons provided 18% of the peak total ankle moment provided by the combined exoskeleton and biological ankle, and 33% of the positive ankle work provided by the combined exoskeleton and biological ankle throughout the gait stride (Fig. 4) during the PoweredEnd condition. As we hypothesized, there were no statistical differences between conditions in joint angles, total moments, or total powers at the ankle, knee, or hip across conditions over the gait cycle (Fig. 5). The Statistical Parametric Mapping (SPM) provided a means to determine if certain phases of the gait cycle had differences in joint kinematics and kinetics, but they were quite similar across all six conditions. There was about 2-degrees greater dorsiflexion during the stance phase in the Boots Only condition compared to the powered exoskeleton conditions. In addition, there was an average 6-degrees greater dorsiflexion during the stance phase in the Deadaptation conditions compared to the powered exoskeleton conditions, but the difference was not statistically significant. Similarly, those conditions had slightly greater peak negative power at the maximum dorsiflexion during stance (∼0.64 W/kg), but the differences were not significant.

**FIGURE 6.**
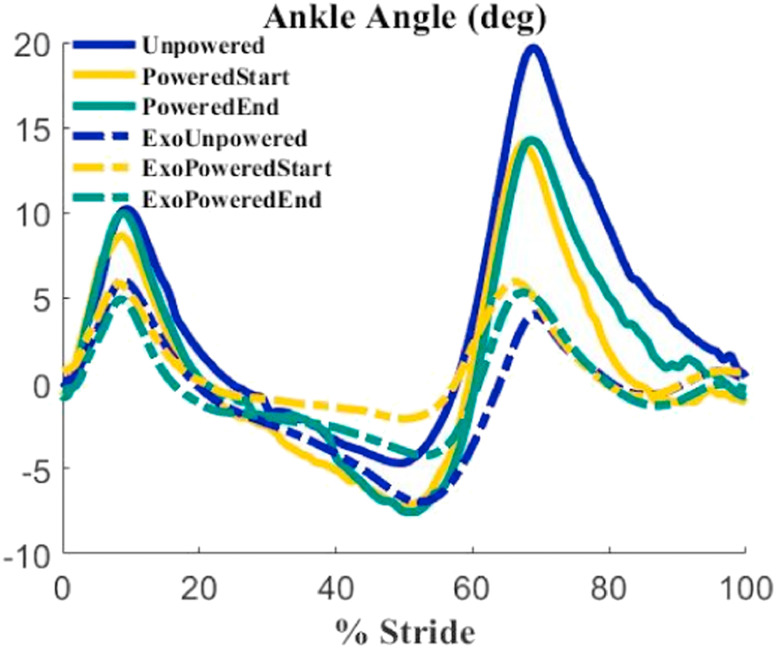
The ankle angle measurements from the exoskeleton (dashed lines) and 3D motion capture (solid lines). There was an average difference of 12 degrees for total range of motion for the unpowered (blue), PoweredStart (Yellow) and PoweredEnd (Green) conditions. Heel contact was at time 0% on the x-axis.

There was a substantial difference between the ankle angle recorded through 3D motion capture and the ankle angle recorded by the exoskeleton software (Fig. 6). There was an average difference of 9 degrees of maximal plantar flexion and 3 degrees of maximal dorsiflexion. This difference results from two factors. First, the biological ankle joint center of rotation did not always match up to the exoskeleton ankle center of rotation. Second, soft tissue compression around the tibia and foot during exoskeleton activation can lead to differences in relative joint measurements for the biological ankle and the exoskeleton angle.

## III. Discussion

In this study we quantified the changes in muscle activity and joint biomechanics of healthy, young participants using a commercially available robotic ankle exoskeleton with a proportional myoelectric controller. As hypothesized, there were no significant changes in joint biomechanics at the ankle, knee, or hip across conditions. During both powered conditions there was a decrease in dorsiflexion and negative ankle power that occurred that may have been caused by the added exoskeleton torque beginning to pull the toe into plantarflexion. We hypothesized that EMG root mean squared values of the triceps surae muscles would reduce after 30 minutes of training compared to the Boots Only condition, but we did not find statistically significant differences. There were slight reductions in muscle activity during stance, but they were not strong enough to affect statistical comparison of root mean squared values during stance. Root mean squared Statistical Parametric Mapping showed a significant decrease in lateral gastrocnemius during terminal stance, and the tibialis anterior during loading from the Boots Only to PoweredEnd conditions, but these differences were short in duration and small in magnitude.

Although some participants had lower triceps surae RMS values (Fig. 3), those changes were washed out by the high variability between participants leading to nonsignificant statistical results. We initially performed a power analysis looking at the change in soleus RMS from the boots only condition to the PoweredEnd condition, as our primary outcome measure, of three participants. In the first three participants we saw a very low standard deviation of 0.0043 at the end of the powered condition, and 0.0126 in the boots only condition. After including all 12 participants, the standard deviation went up 0.0213 in the PoweredEnd condition and 0.0231 in the boots only condition. The initial effect size was 0.97 for a total of 8 subjects. Whereas the post hoc analysis showed an effect size of 0.36 with a required sample size of 45 participants. One defining factor of proportional myoelectric control is the ability of the user to have direct neural control of the exoskeleton. For this reason, larger variability in outcome measures is not unexpected because the exoskeleton response is more personalized. However, in past studies, the use of pneumatic actuators which have a much smaller bandwidth and provided larger assistive torques than the electromechanical actuators used here, led to significant decreases in EMG RMS values [6], [16], [17], [20], [21]. In addition, the lower percent change in EMG RMS on the final day of testing, while the amount of torque supplied by the exoskeleton stayed at a similar level to the first day of training, may indicate that individuals were using the assistance of the device more efficiently by maintaining their EMG at a similar magnitude with and without the exoskeleton. Rather than doing less work by using the exoskeleton, they were keeping the torque and EMG at higher levels to augment their walking.

Fig. 3 shows the variable EMG RMS activation across participants on the first and fourth day of training. It is important to recognize that two participants in particular have very large increases in lateral gastrocnemius RMS in the PoweredEnd of the Fourth day compared to the boots only condition on that day. One explanation for this is that these participants used their lateral gastrocnemius to stabilize the leg, and prevent the knee from buckling, while the torso is being propelled forward using the assistance from the exoskeleton [22], [23]. This may have allowed these participants to leverage the work done at the ankle by the exoskeleton without destabilizing their gait. This led to a prolonged activation of the lateral gastrocnemius, and thus a larger RMS value compared to other participants. Fig. 3 further demonstrates what previous work with exoskeletons has shown, that participants differ in how they activate their muscles when adapting to external assistance [17], [24].

One possible explanation of our findings is that the relatively low mechanical power output of the exoskeleton resulted in a lower-than-expected EMG root mean squared reduction. The magnitude of the ankle exoskeleton mechanical power from the Dephy Exoboots was well below what has been found for pneumatic, tethered ankle exoskeletons [6], [16], [17], [20] and electromechanical ankle exoskeletons using Bowden cables to deliver mechanical power to the ankle from actuators removed from the ankle [25], [26]. We found a peak power of 0.63 W/kg in our study. In comparison, the other studies have values ranging from 1.1 W/kg to 2.7 W/kg. This suggests that the ankle exoskeletons we used are on the far end of low mechanical power.

**FIGURE 7.**
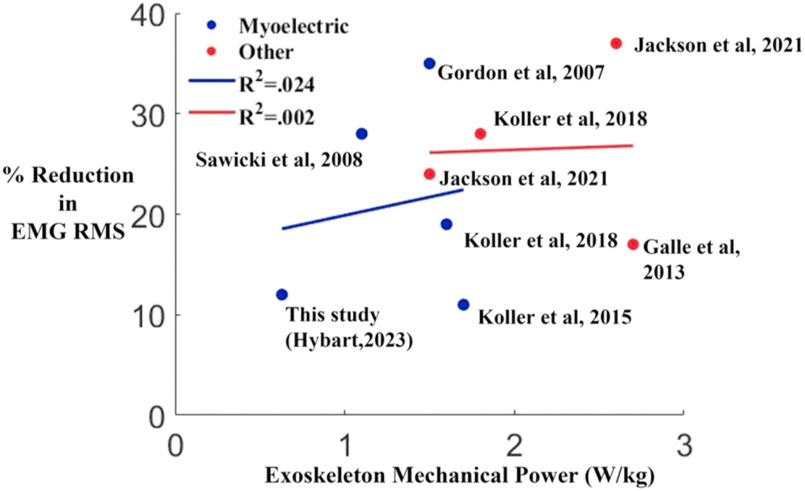
The exoskeleton mechanical power (W/kg) versus the percent reduction in EMG root mean squared at the end of adaptation in eight different studies. Five studies, including the one presented in this paper, using proportional myoelectric control (Blue). Three studies using state-based exoskeleton control methods (Red). There was not a strong linear relationship between increased power output and increased reduction in EMG root mean squared when using state-based controller (R^2^ = 0.002).

Comparing triceps surae muscle activity changes in healthy, young participants using robotic ankle exoskeletons, suggests there are many factors beyond exoskeleton power that influence muscle activity reductions. In a review of data from previous literature, we found there is not a strong correlation between exoskeleton mechanical power output and EMG reduction. Fig. 7 shows the exoskeleton mechanical power (W/kg) versus the percent reduction of EMG root mean squared from baseline to the final timepoint in several studies. When using a proportional myoelectric controller, the relationship between relative power output from the exoskeleton and EMG reduction is not strongly correlated. An increase in exoskeleton power does not strongly indicate more reduction in EMG activation (R^2^=0.024). Similarly, controllers using motor position and/or gait phase timing to determine timing of assistance, did not see a linear increase in EMG reduction as exoskeleton power increased (R^2^=0.002) [6], [7], [16], [17], [20], [25]. We saw a lower power output than other studies using proportional myoelectric control, with our exoskeleton supplying 29% of total ankle power compared to an average of 52% in studies using pneumatic muscles. We also saw a 12% decrease in EMG root mean squared compared to an average of 23% in studies using pneumatic muscles. It seems that the exoskeleton hardware design and nuances of the controllers may have a large effect on how ankle exoskeleton mechanical power assistance affects user muscle activity. In the future, finding a way to increase the power output of the exoskeleton may increase the reduction in EMG root mean squared during adaptation, but a higher exoskeleton mechanical power does not guarantee an increased reduction in EMG root mean squared.

Other papers that have studied neuromuscular adaptation or metabolic benefit of the Dephy ExoBoots used various types of controllers with typically better outcomes than seen in this article. Two studies using the Dephy ExoBoots under different state-based controllers recorded metabolic cost of transport [27], [28]. Medrano et al. (2022) used a current based controller to apply plantarflexor torques with varying onset times, magnitudes, and durations to determine perceivable metabolic cost changes. Participants in this study walked at 1.25 m s^−1^. The average metabolic cost reduction they achieved was 12.6% compared to the average metabolic cost of walking without an exoskeleton. They reported peak exoskeleton torques of between 12 and 20 Nm. Shepherd et al. (2022) used a convolutional neural network gait phase estimator to provide a peak torque at 84% of stance phase at variable walking speeds. Participants in this study walked at the sinusoidally varying speeds of 1.1 to 1.6 m s^−1^ with a 30 s period. The average reduction in metabolic cost they achieved was 5.2% during variable speed walking compared to not wearing the exoskeleton. They reported a peak exoskeleton torque of 30 Nm. A third study by Acosta-Sojo and Stirling (2022) reported inter-subject variability in EMG RMS when learning to walk with the Dephy ExoBoots. In this study they reported peak torques of ∼17–27 Nm [24]. Comparatively, we saw torques between 7 and 17 Nm across individuals. Our exoskeleton torque is on the lower end of what other studies have achieved using the same hardware, which may be due to the hardware version and different current limits. Another cause for our nonsignificant changes in EMG RMS might be that our controller applied torques earlier in the gait cycle and over a longer duration than the other two papers. Medrano et al. (2022) had a control duration of 10–60% of the gait cycle with an onset at 25–50% of the stride. Shepherd et al. (2022) applied torques for 38% of the stride with an onset at about 30%. Peng, et al. (2022) showed analyzed data on torques with an onset of 22–29% of the gait cycle and a duration of ∼30% of the stride [29]. Our applied torque onset was earlier and stayed on longer than the other studies using the Dephy ExoBoots, with our onset being at 10% of the gait cycle, and the duration being 55–60% of the stride on average. An earlier onset is typical in exoskeletons under proportional myoelectric control because it is directly responding to the physiological signals. The triceps surae muscles are active throughout the stance phase [16], [17]. The use of proportional myoelectric control in combination with a hardware system that has a nonlinear relationship between muscle input and torque output may lead to a non-ideal relationship between onset timing, duration, and magnitude of actuation. Future implementations of proportional myoelectric control with electromechanical motor could employ state-based gains to correct this nonlinear issue.

Most robotic ankle exoskeleton papers report ankle angle for each condition, but do not separately report the exoskeleton ankle angle. Fig. 6 shows that the ankle angle measurements from the exoskeleton and 3D motion capture do not show the same total range of motion. In the unpowered condition the ankle moved from 5 degrees of dorsiflexion to 20 degrees of plantarflexion, but the exoskeleton reported 7 degrees of dorsiflexion to 4 degrees plantarflexion. In the PoweredStart and PoweredEnd condition the ankle moved from 8 degrees of dorsiflexion to 14 degrees of plantarflexion, but the exoskeleton reported 2.5 degrees of dorsiflexion for both conditions and 6 and 7 degrees of plantarflexion respectively. This is an average difference of 13 degrees from the total ankle angle from motion capture compared to the reported angle from the exoskeleton. This difference in reported angles is due in part to the exoskeleton interfacing with the human at the shin via a cuff attached to the shank with an elastic strap. There is some research on how the effects of the physical interface between the human and exoskeleton affects transmission of power [30], [31]. The combination of soft tissue deformation of the calf and stretch at the elastic cuff means that the exoskeleton moves separately from the user at different points in the gait cycle. In addition, exoskeleton joint movement relative to biological ankle joint movement may lead to differing range of motions [32]. The exoskeleton is rigidly attached to the shoe, whereas the human ankle joint is not. This means that within the shoe the ankle joint center of rotation may shift compared to the exoskeleton joint center. If the center of rotation of the exoskeleton joint and biological ankle joint are moving this much with respect to one another, this may lead to loss of translation of power from the exoskeleton to the user. If the exoskeleton angle had matched the biological ankle angle, then the power and positive work would have been 15% and 35% higher assuming the same moment profile. This demonstrates that there was a major loss of mechanical assistance due to movement of the exoskeleton center of rotation relative to the ankle center of rotation.

This study was the first study to use proportional myoelectric control using a portable electromechanical ankle exoskeleton instead of a pneumatic ankle exoskeleton. We were not able to produce the same amount of power output from the exoskeleton compared to those using pneumatic muscles. However, a benefit of using a portable device is that the next steps include real world use of the exoskeletons. We want to compare treadmill compared to outdoor walking because one proposed benefit of proportional myoelectric control is the ability to adapt to changing environments in the real world. Real world walking was not possible with pneumatic exoskeletons. For this reason, we believe that even though we did not achieve significant decreases in EMG root mean squared, this study shows that it is possible to implement this type of control on an electromechanical ankle exoskeleton. Myoelectric control may have a valuable application in rehabilitation as it would amplify errors in muscle recruitment and may improve the user's internal model [5], [33]. Amplifying errors allows the user to understand more clearly where they can improve neuromuscular control. This study shows that it is possible to implement proportional myoelectric control on a portable device for future rehabilitation research.

There were several limitations to this study. One limitation is that we did not record hip flexor muscle activity. Previous studies showed reductions in both triceps surae and rectus femoris EMG root mean squared with adaptation to a robotic ankle exoskeleton under proportional myoelectric control. Understanding changes in EMG in the hip flexors may give more insight into how the participants interacted with the exoskeletons. Another limitation is our sample size. Variability in EMG activation patterns during adaptation to ankle exoskeletons means a larger sample size may be necessary to see a significant reduction in EMG root mean squared [24]. However, the Standardized Mean Difference values for the triceps surae EMG root mean squared indicate that the differences were very small even if they were real. The value of 0.3 is considered small by most statistical standards, and our Standardized Mean Differences for the soleus (0.12), medial gastrocnemius (0.05), and lateral gastrocnemius (0.17) fell well below that value [34]. A final limitation of this study was the low mechanical power output of the exoskeleton. The control mechanism we used altered the input current to the exoskeleton proportionally to the user's muscle activation. In pilot trials, we attempted to increase the maximum current into the exoskeleton to boost the mechanical power at push-off. However, we encountered safety limits from the actuator that prevented us from achieving higher torques.

Proportional myoelectric control may have benefits in rehabilitation after incomplete spinal cord injury and stroke [35]. It essentially strengthens the joint mechanics such that a little muscle activation produces a greater joint torque than would normally occur. In theory, this is similar to placing a rehabilitation patient in a pool of water so that buoyancy counteracts gravity [36]. Allowing a small motor command to produce a large joint motion (and greater proprioceptive feedback signal) could promote Hebbian learning [37]. Proportional myoelectric control of ankle exoskeletons can promote substantial changes in muscle recruitment in individuals with incomplete spinal cord injury over a time span of 24 minutes [35]. Electromechanical lower limb exoskeletons like the Dephy ExoBoot should facilitate additional clinical research studies given their portability.

## IV. Conclusion

We tested the hypotheses that individuals using electromechanical ankle exoskeletons with proportional myoelectric control would: 1) maintain baseline joint biomechanics and 2) reduce triceps surae EMG root mean squared values after a 30-minute adaptation period. Participants did not change their joint biomechanics across conditions. There were small but non-significant decreases in lateral gastrocnemius, medial gastrocnemius, and soleus EMG root mean squared. The result may have been because of the low mechanical power output of the ankle exoskeletons, but comparison to previous research suggests that there is not a direct correlation between ankle exoskeleton mechanical power and triceps surae muscle activity changes. Applying neural based controls to portable robotic lower limb exoskeletons is an important step towards using them as rehabilitation aids for individuals with neurological disabilities.

## V. Materials and Methods

We recruited 12 healthy participants (5 female, 7 male) with the following characteristics: age 22.6 (8.66) yrs.; height 1.73 (0.751) m; and body mass 69.3 (10.2) kg; mean (s.d.). Participants had no previous neurological or musculoskeletal conditions (Table 1). Each participant was right-handed and had no previous experience walking with a robotic ankle exoskeleton. Participants read and signed an informed consent form approved by the University of Florida Institutional Review Board (IRB# 201801218). We expected 12 participants would provide sufficient statistical power based on our prior research using similar methods and controller for testing of pneumatic ankle exoskeletons [6], [16]. After collecting pilot data on three participants, a preliminary statistical power analysis indicated 10 participants should provide 0.8 power for an alpha equal to 0.05. The mean characteristics of the participants in the preliminary statistical power analysis (2M,1F) were: age = 22, weight = 73.6, height = 1.74, walking speed = 1.03.

**TABLE 1:** Participant Characteristics

Participant Number	Age	Gender	Height (m)	Weight (kg)	Walking Speed (m/s)
1	20	F	1.60	73.5	0.95
2	22	M	1.78	74.8	1.20
3	24	M	1.83	72.6	0.95
4	18	F	1.67	59.9	1.00
5	18	M	1.78	61.2	1.20
6	18	M	1.70	79.4	1.20
7	18	M	1.78	90.7	1.15
8	18	F	1.75	52.6	1.15
9	18	M	1.75	58.1	1.35
10	26	F	1.73	74.8	1.30
11	21	M	1.80	63.5	1.30
12	50	F	1.57	70.3	1.10

We used the commercially available Dephy ankle exoskeletons with the open-source software option, allowing us to implement our own high-level control on their hardware (Dephy, Inc. Maynard, MA). A proportional-myoelectric controller with an input of the user's soleus muscle provided the input signal to the motor [38]. The soleus electromyography (EMG) data were high-pass filtered (2nd order Butterworth, cutoff frequency 50 Hz), full wave rectified, and low-pass filtered (2nd order Butterworth, cutoff frequency 8 Hz) (Fig. 9). The resulting signal was then multiplied by a subject specific gain to reach the desired maximum current. Subject specific gains were static gains determined during the Unpowered condition, and ensured the controlling current reached the maximum value of 7.6 A q-axis (phase) current, which is equivalent to 20 A of line-to-line current as described by Dephy. The controlling current was transformed via a variable transmission ratio into an ankle torque that pulled the toe down. The exoskeletons only assisted in plantarflexion and were transparent during swing. In summary, the controller uses filtered EMG signals to determine the amount of torque provided at the exoskeleton ankle joint. The entire system including a raspberry pi in a chest pack added 4.5 kg to the participant.

Bipolar skin electrodes over the soleus, medial and lateral gastrocnemii, and tibialis anterior (Coapt, Inc. Chicago, IL) recorded the EMG data. We only used the soleus EMG in the control software for the exoskeletons. We collected lower body motion capture using the Rizzoli Lower Limb markerset using a 20-camera Optitrak system (NaturalPoint, Inc. Corvallis, OR), and computed inverse dynamics in Visual 3D. We analyzed data from the right lower limb for all exoskeleton and biological kinematics and kinetics. Participants were all self-reported right-leg dominant.

**FIGURE 8.**
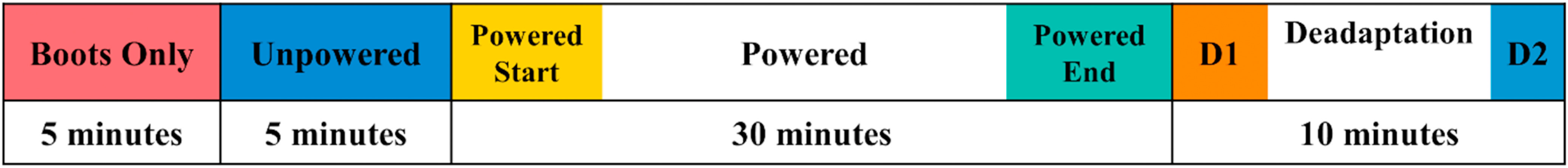
Timeline of the protocol, with powered and deadaptation phases highlighted. Participants walked for a total of 50 minutes. PoweredStart was the first 30 strides after turning the exoskeleton on and PoweredEnd was the final 30 strides before turning the exoskeleton off. D1 was the first 30 strides after turning off the exoskeleton after practice and D2 was the final 30 strides of walking with the exoskeleton off at the end of the experiment.

For the exoskeleton data collection, participants walked at a self-selected speed (1.15±0.13 m/s) on a force instrumented treadmill (Bertec, Corp. Columbus, OH). Prior to donning the exoskeletons, participants walked overground for 10 meters to determine their preferred overground walking speed. We averaged together three trials to determine their preferred speed. Participants first walked for 5 minutes with only the boots of the exoskeletons on (Boots only condition). We then attached the exoskeletons to the boots and participants walked for 5 minutes with the exoskeletons on but not actuating (Unpowered condition). During this condition, we determined the participant specific gains and thresholds for the controller. Then the participants walked for a total of 30 minutes, broken up into three 10-minute periods, with the exoskeleton powered (Powered condition). Finally, we powered off the exoskeletons, and the participants underwent a 10-minute deadaptation period (Deadaptation condition) (Fig. 8). Between each condition the participants rested as needed but were asked not to take any extraneous steps while resting. Although this article focuses on initial adaptation to the exoskeletons, participants returned for three more training sessions. By the end of the fourth day participants had walked ∼106 minutes with the exoskeletons powered. On the fourth day we measured 6 minutes of walking on the treadmill with and without the exoskeleton at the same speed as day one. We did not collect motion capture data, and only present the subject specific EMG RMS and exoskeleton torque data here.

**FIGURE 9.**
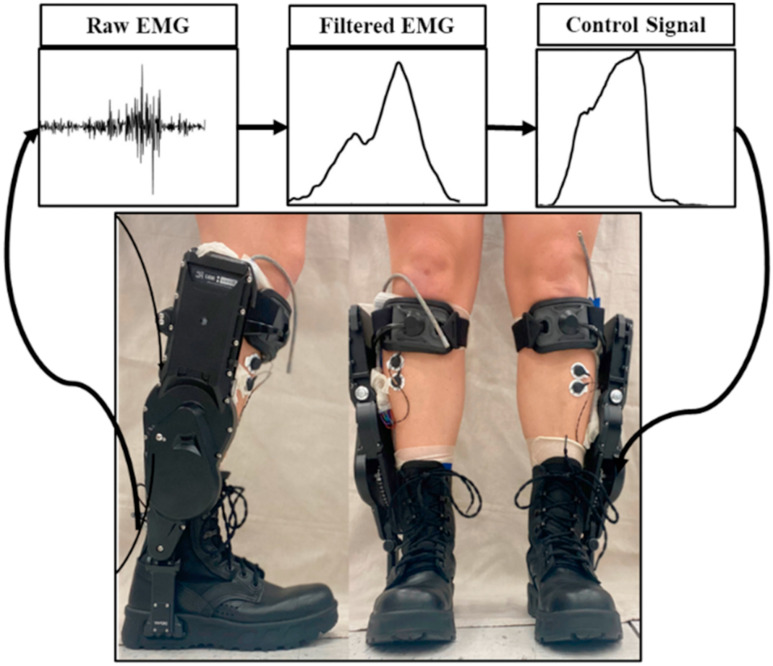
Dephy Exoboots on an individual, with Coapt EMG sensors on the tibialis anterior exposed. Example raw and filtered EMG from the soleus, as well as an example control signal which applies a plantar flexor torque during push-off.

For each condition we averaged 30 strides at the desired time points and compared muscle activity and kinematics [39]. These time points included the last 30 strides of Boots Only, Unpowered, Powered (PoweredEnd) and Deadaptation (Deadaptation2, D2), as well as the first 30 strides of the Powered (PoweredStart) and Deadaptation (Deadaptation1, D1) conditions. For EMG activity data were normalized to the maximum value in the Boots Only condition for each person before averaging. The moment, mechanical power, and mechanical work data were normalized to bodyweight for each participant.

We used SPSS Statistics Software (IBM) to perform a 1-way ANOVA on the root mean squared of the EMG data during stance phase (or swing phase for the Tibialis Anterior) to determine the effects of condition on root mean squared. In addition, we performed a 1-way ANOVA on the timeseries EMG and joint biomechanics data using Statistical Parametric Mapping (SPM) to determine if there were specific timepoints in the gait cycle that the average EMG or average joint biomechanics were significantly different. When we found a significant effect (p<0.05) in either, we used post-hoc Tukey HSD method to determine between which means there were significant differences. We calculated the Standardized Mean Difference for the triceps surae muscle root mean squared by dividing the difference in means of the EMG root mean squared of the Boots Only and PoweredEnd conditions by the mean standard deviation of the EMG root mean squared of both conditions. A Standardized Mean Difference of 0.3 is considered low.
